# Virulence Factors of *Clostridioides* (*Clostridium*) *difficile* Linked to Recurrent Infections

**DOI:** 10.1155/2019/7127850

**Published:** 2019-12-24

**Authors:** Laura Tijerina-Rodríguez, Licet Villarreal-Treviño, Rayo Morfín-Otero, Adrián Camacho-Ortíz, E. Garza-González

**Affiliations:** ^1^Departamento de Microbiología e Inmunología, Facultad de Ciencias Biológicas, Universidad Autónoma de Nuevo León, San Nicolás de los Garza, Mexico; ^2^Centro Universitario de Ciencias de la Salud, Universidad de Guadalajara, Hospital Civil de Guadalajara “Fray Antonio Alcalde” e Instituto de Patología Infecciosa y Experimental, Guadalajara, Mexico; ^3^Hospital Universitario “Dr. José Eleuterio González”, Universidad Autónoma de Nuevo León, Monterrey, Mexico

## Abstract

From 20 to 30% of *Clostridioides* (*Clostridium*) *difficile* infection (CDI), patients might develop recurrence of the infection (RCDI) and, after the first recurrence, the risk of further episodes increases up to 60%. Several bacterial virulence factors have been associated with RCDI, including the elevated production of toxins A and B, the presence of a binary toxin CDT, and mutations in the negative regulator of toxin expression, *tcdC*. Additional factors have shown to regulate toxin production and virulence in *C. difficile* in RCDI, including the accessory-gene regulator *agr*, which acts as a positive switch for toxin transcription. Furthermore, adhesion and motility-associated factors, such as Cwp84, SlpA, and flagella, have shown to increase the adhesion efficiency to host epithelia, cell internalization, and the formation of biofilm. Finally, biofilm confers to *C. difficile* protection from antibiotics and acts as a reservoir for spores that allow the persistence of the infection in the host. In this review, we describe the key virulence factors of *C. difficile* that have been associated with recurrent infections.

## 1. Introduction

*Clostridioides* (*Clostridium*) *difficile* infection (CDI) is the leading cause of healthcare-associated diarrhea worldwide. In 2011, increased CDI was reported in the United States by the Centers for Disease Control with an estimated 453 000 infections (83 000 had at least one recurrence) and 29 000 deaths [1].

CDI severity has increased in the last decade with outbreaks in the United States, Canada, the United Kingdom, Western Europe, Japan, Korea, China, Hong Kong, and some countries of Latin America. This increased severity has been coincident with the spread of the epidemic strain designated as North American Pulsed (NAP)-field type 01, restriction endonuclease analysis (REA) as group BI, (BI/RT-027/BI), and polymerase chain reaction (PCR) ribotype (RT) 027 (NAP1/BI/027) [2–8].

From 2008 to 2014, CDI cases declined considerably in the United Kingdom, from 55,498 to 13,361, as a result of a surveillance scheme implemented by the National Health Service, including antibiotic stewardship, improvement protocols for infection control in hospitals, and the creation of the *C. difficile* ribotyping network in aims to prevent CDI transmission and control epidemic strains [9–13].

However, CDI is not only of worldwide concern due to ribotype 027 but also due to the emergence of other virulent strains, including ribotypes 027, 078, 001, 176, 020, 002, and 106, in many populations [1, 14–17].

## 2. Recurrence of CDI

Primary CDI is predominantly treated with standard antibiotic therapy, including metronidazole, vancomycin, and fidaxomicin, the more recently FDA-approved drug, depending on severity [18]. Nevertheless, 20–35% of patients may develop the recurrence of symptoms, which is defined as a recurrent infection (RCDI) [19–23]. After the first recurrent episode, patients are more likely to have subsequent recurrences, and by the third episode, risk of recurrence can reach 60% [24, 25]. Several studies have evaluated administration of fidaxomicin versus vancomycin and metronidazole for RCDI patients, with lower recurrent episodes and fewer deaths for fidaxomicin [18, 26, 27].

## 3. Relapse and Reinfection

RCDI may occur due to relapse, defined as the persistence of the same strain causing the initial infection, or reinfection, defined as the acquisition of a genotypically distinct *C. difficile* strain from an exogenous source [28]. Furthermore, patients with ribotype 027 strains present a higher risk of relapse than those with other ribotypes [7].

## 4. Ribotypes Associated with Relapse or Reinfection

The glycosylating toxins, toxin A (TcdA) and toxin B (TcdB), are primarily responsible for the symptoms associated with CDI and are the key mediators of pathogenesis [29]. These toxins have been shown to bind to the cell surface and translocate to the cytosol of the host epithelial cells where they glycosylate and inactivate important GTPases (including Rho, Rac, and Cdc42), leading to actin cytoskeleton alternations, cell rounding, apoptosis, and cell death [30, 31].

Several studies have shown elevated sporulation rates in epidemic strains, including the hypervirulent NAP1/BI/027 strain [32]. Also, these strains have been found to contain increased levels of toxins, which are associated with deletions in the toxin negative regulator *tcdC* (18 bp and 39 bp deletions for the 027 and 078 strains, respectively) in *in vitro* models [33, 34]. However, in more complex models, the 027 strain has been shown to have a longer growth cycle, where toxin production starts slightly earlier than that of other strains, and toxins tend to accumulate [35, 36].

Hypervirulent *C. difficile* also produces a third toxin called binary *C. difficile* toxin (CDT). CDT is a transferase that can irreversibly ADP-ribosylate actin and promote disruption of the actin cytoskeleton [31]. The presence of CDT and mutations in *tcdC* increases the risk of RCDI (odds ratio (OR), 5.3; 95% confidence interval (CI), 3.52–6.09) (Table 1) [2, 64].

RCDI is more frequent in patients infected with the 027 strain than in those infected with non-027 strains (*P* < 0.001). Besides, the clinical cure rate has been reported to be lower in 027-infected patients than in those with non-027 infections when treated with fidaxomicin (*P*=0.007) or vancomycin (*P*=0.02) [3].

## 5. Antibody Response to Toxin A

In several studies, the immune response to toxins A and B has been described, with higher titers of immunoglobulins IgG antitoxin A in asymptomatic carriers than noncarriers, and higher titers of IgG and IgM against toxin A and toxin B, but regression analysis showed significance for recurrent infection and low antitoxin A for 027 and primarily for all types with patients with little antitoxin B (*P*=0.02) [65, 66].

Patients with a single episode of CDI had higher concentrations of serum IgM against toxin A on day 3 of initial CDI than those with RCDI (*n* = 22; *P*=0.004). On day 12, patients who had a single episode of diarrhea (*n* = 7) had higher serum IgG values against toxin A than those with RCDI (*n* = 9; *p*=0.009). IgG response to toxin A (12 days after onset of CDI) during an initial episode confers protection against recurrence (OR, 48.0; 95% CI, 3.5–663) [25]. Nevertheless, CDI patients who received neutralizing antibodies against toxin A showed no difference in the frequency of recurrence in comparison with CDI patients receiving placebo (17% and 18%, respectively, *P* = NS) [67].

## 6. Biofilm Production

Bacterial biofilms are associated with antimicrobial resistance, act as a survival niche, and protect bacteria, which can be in a dormant form with prolonged growth rates deep within the biofilm structure. Biofilms have been reported for several *Clostridium* species, including *C. perfringens*, *C. thermocellum*, and *C. acetobutylicum* [68, 69]. Similarly, *C. difficile* growth has shown well-organized communities on abiotic surfaces and well-structured biofilms *in vitro* and *in vivo* [42, 61, 70], with differences in the level of biofilm production between some strains in monoculture biofilms [42, 71]. In addition, a range of studies has characterized the supernatant and the polymeric composition and architecture of the biofilm matrix in *in vivo* and *in vitro* models, which is composed of extracellular DNA, polysaccharides, and proteins similar to *B. subtilis* biofilm [42, 54, 61, 72].

Notably, in a chemostat gut model, *C. difficile* (vegetative and spore forms) has been shown to participate in multispecies communities forming a robust biofilm that accumulates toxins. In addition, this biofilm is a potential reservoir for the reestablishment of *C. difficile* after primary antimicrobial therapy has finished, when gut levels of antimicrobials are at subminimal inhibitory concentration [36, 43, 44]. Furthermore, the biofilm matrix showed a preferential localization of spores that have a higher resistance to some antibiotics (metronidazole and vancomycin) (Table 1). Taken together, these observations may explain the long-term persistence of strains involved in primary and/or recurrent CDI [42, 45].

## 7. Regulation by Quorum Sensing

Quorum sensing (QS) is the regulation of gene expression of virulence factors (biofilm production, attachment, motility, toxin production, and sporulation) in response to environmental changes due to cell-to-cell communication. It is mediated by small diffuse molecules known as autoinducers produced by individual bacteria. The level of autoinducers is cell-density dependent: when the density is high, autoinducers are detected by other bacteria, enabling them to coordinate physiological activities [38, 73, 74].

Orthologues of the accessory-gene regulator (Agr) ACDB, the global regulatory locus that encodes AgrA, have been found within the genome of most *C. difficile* isolates, including the hypervirulent strain 027/BI/NAP1. AgrA has critical roles in controlling gene expression and enhancing the production of colonization factors and exoproteins essential for the pathogenic process [38, 39]. Furthermore, it is the transcriptional regulator of the best-understood QS system in Gram-positive bacteria, including *Staphylococcus aureus* [39].

*C. difficile* production of toxins A and B are controlled by an Agr-quorum signaling system mediated through a small thiolactone that can be detected in stools of CDI patients [40]. Some strains encode two Agr loci in their genomes (*ag1* and *agr2*), with the first being present in all strains and the second being present in a few strains. It has been shown that the *agr1* mutant cannot produce both toxins and that toxin production can be restored with the wild type *agr1.* Furthermore, it has been demonstrated that the *agr1* mutant can colonize but cannot cause disease in a murine CDI model (Table 1) [39, 75].

## 8. Sporulation and Germination

Another key virulence factor involved in *C. difficile* pathogenesis and colonization is spore production, with differences in germination rates being lower in spores from biofilm than those from a vegetative culture [45]. Spore production is mediated by the master regulator of the sporulation pathway (*spo0A*). In mice infected with sporulating and nonsporulating *C. difficile* strains (*spo0A* mutants), no recurrence of CDI was found after vancomycin treatment, and the *spo0A* mutant infection was not transmissible between hosts [59]. *Spo0A* mutants are associated with defective biofilm formation and low sporulation in biofilms (0.0001%), suggesting an essential link between *Spo0A* and biofilm production, such as that seen in *Bacillus subtilis* [42, 61, 62].

The production and accumulation of spores within *C. difficile* biofilms are likely to be significantly associated with RCDI, with further germination of vegetative toxin-producing cells after cessation of antibiotic therapy. Metabolically dormant spore forms can protect *C. difficile* from adverse conditions, such as nutrient starvation, antimicrobial agents, disinfectants, heat, and desiccation, and help the bacteria survive attacks of phagocytic cells [76]. Furthermore, antibiotic treatment triggers the excretion of higher sporulation of *C. difficile* in mice. Therefore, in most cases, when antibiotic therapy is stopped, a recovery occurs and the super-shedder state of *C. difficile* is suppressed [77].

A novel exosporial layer has been found in spores from biofilms, composed of fine fibers and darkly staining granules. This layer is surrounded by a thin layer and is acquired after mother cell lysis; it has been found in 027 strains associated with multiple recurrent episodes of CDI (Table 1) [45, 59, 60]. The specific role of these structural differences of the exosporium in spores is not clear. A previous study showed that the *C. difficile* R20291 strain (RT-027) showed higher affinity to the host cell membrane and microvilli of intestinal epithelial cells [78], suggesting that the differences in composition of the exosporium of *C. difficile* spores might regulate the adherence to intestinal cells of the host (Table 1) [78–80]. Additional studies are needed to determine the role of the structural properties of the *C. difficile* layer and spore exosporium for the development of recurrent infection.

## 9. Adhesion Factors

Several nontoxin factors involved in the virulence and infection processes have been described, including surface proteins (cell wall and surface layer (S-layer) proteins), pili, flagellin, flagellar cap, and fibronectin-binding proteins [42, 53].

The S-layer protein A (SlpA) is the predominant outer surface and has shown to be the major contributor of *C. difficile* adherence to epithelial cells *in vitro*. SlpA is cleaved after translation in high and low molecular weight (HMW and LMW) subunits for the assembly of the paracrystalline layer. Interstrain sequence variability of LMW subunits has been associated with higher adhesion efficiency in hypervirulent strains [48–51].

The cell wall protein 84 (Cwp84) is one of the primary proteases that is exported by the cell and cleave several adhesins such as SlpA for the assembly of the paracrystalline layer and the degradation of extracellular matrix proteins (fibronectin, laminin, and vitronectin). This degradation triggers the release and dissemination of *C. difficile* in the host, which are related to the recurrence of infection (Table 1) [49, 81–83].

Cwp84 is present in all *C. difficile* strains, and those with the highest proteolytic activity are associated with stronger adhesion and production of thicker biofilm, planktonic growth defect, and virulence *in vivo* [49, 72]. In addition, a recent study found overexpression of *cwp84* in a biofilm model from recurrence causing strains, this phenomenon was not observed in the biofilm produced by nonrecurrent strains [84], suggesting an association with recurrent infection.

SlpA cleavage could be accomplished by other proteases in the absence of *cwp84*, such as *cwpV*, *cwp66*, and *cwp13* [49, 52]. These findings suggest an essential role of some surface proteins associated with increased host-pathogen adherence, which may be related to the maintenance of CDI.

In addition to propulsion, motility components provide bacteria with other advantages, such as adherence and cell internalization. *C. difficile* possesses peritrichous flagella, which induce the adhesion and establishment of the bacteria including the strains without complete and functional flagella as a result of mutations [56, 57, 85–87]. The filament from *C. difficile* flagella is mostly composed of single flagellin subunits and flagellar cap proteins, both of which are modified posttranslationally. Therefore, as a consequence of a noncomplete functional flagella, these components do not confer motility but enhance binding of *C. difficile* to abiotic surfaces, as well as reduced biofilm formation, leading to the attenuation of colonization and relapse *in vivo*, suggesting a role of flagella in the process of adherence and biofilm formation independent to motility (Table 1) [42, 56, 57].

## 10. Concluding Remarks

RCDI development is associated with hypervirulent strains and may be attributed to a high rate of sporulation and the maintenance of spores encased in a *C. difficile* biofilm, which is resistant to antibiotic therapies. Besides, several adhesion-related proteins are involved in RCDI development and the establishment of the infection.

Antibiotics have been demonstrated to disrupt colonic microbiota, placing the patient at a high risk of further recurrent episodes. Further studies on RCDI development are needed to assess the correlation of these potential virulence traits and the persistence of *C. difficile* infection.

## Figures and Tables

**Table 1:** Summary of presumptive virulence factors associated with recurrent *C. difficile* infections.

Factor	Mechanism/function	Risk/association	Source
*tcdC* and binary toxin	Production of elevated toxin A and B levels in hypervirulent strains	Increased pathogenicity *in vivo* and *in vitro*	[37]

*agr1* locus (accessory-gene regulator)	Positive regulation of toxin A and toxin B production, independent of *tcdC*	Regulation of virulence, associated with increased colonization	[38–41]

Biofilm	Survival niche of *C. difficile* with multispecies communities Accumulation of toxins and biomass in variant strains regulated by quorum sensing	Long persistence/protection of *C. difficile*	[36, 42–46]
	Accumulation of spores	Reduced susceptibility to antibiotics	[45, 47]

SlpA (S-layer protein A)	Presence and low molecular weight subunits with sequence variability in hypervirulent strains	Increased adhesion to gut mucosa	[48–52]

Cwp84 (cell wall protein 84)	Cleavage of adhesins, such as SlpA, for the paracrystalline layer assembly	Release and dissemination of *C. difficile* in the host	[49, 52]
	Degradation of several extracellular matrix proteins (fibronectin, laminin, vitronectin)	Increase adhesion and colonization	[48, 49, 53]
	Production of thicker biofilm in strains with high proteolytic activity associated to Cwp84	Enhanced virulence and host-pathogen adherence; maintenance of CDI	[54, 55]

Flagella	Presence of posttranscriptional modifications in flagellin and flagellar cap proteins	Increased biofilm, adherence, and cell internalization, associated with efficient colonization *in vivo*	[42, 56, 57] [58]

Spores	Development of structural morphotypes of outermost exosporium layers (thin or thick)	Associated to host-spore interactions, dfferences in affinity to epithelial cells	[12, 45, 59, 60]
	Expression of the sporulating regulator *spo0A* is associated with high spore production and biofilm formation	Transmission of CDI and maintenance of *C. difficile* in the host, despite the antibiotic treatment	[42, 45, 59, 61–63]
